# Superconductivity in topological semimetals

**DOI:** 10.1093/nsr/nwy155

**Published:** 2018-12-10

**Authors:** Jian Wang

**Affiliations:** 1 International Center for Quantum Materials, School of Physics, Peking University, China; 2 Collaborative Innovation Center of Quantum Matter, China; 3 CAS Center for Excellence in Topological Quantum Computation, University of Chinese Academy of Sciences, China; 4 Beijing Academy of Quantum Information Sciences, China

Topological superconductors, where the bulk state shows superconducting gap and Majorana fermions constitute gapless edge states, have become one of the most important topics in physical sciences. Majorana fermions are their own antiparticles and Majorana quasiparticles in a solid-state system obey non-Abelian statistics, and thus can be used for fault-tolerant topological quantum computation.

Due to both scientific and applicable values, plenty of effort has been made in searching for topological superconductors. The major effort is designing heterostructures composed of Bardeen–Cooper–Schrieffer (BCS) superconductor-topological insulator or BCS superconductor–spin orbit coupling material, where the superconducting proximity effect makes the interface or junction become an effective topological superconductor [1–5]. Then, with applying magnetic field, the zero bias conductance peak (ZBCP), a typical signature of the Majorana-bound state, has been observed at the ends of nanowires or vortex cores by tunneling measurements [6–8]. Nevertheless, there is still a lot of debate over whether the ZBCP in these measurements is truly from the Majorana-bound state.

**Figure 1.**
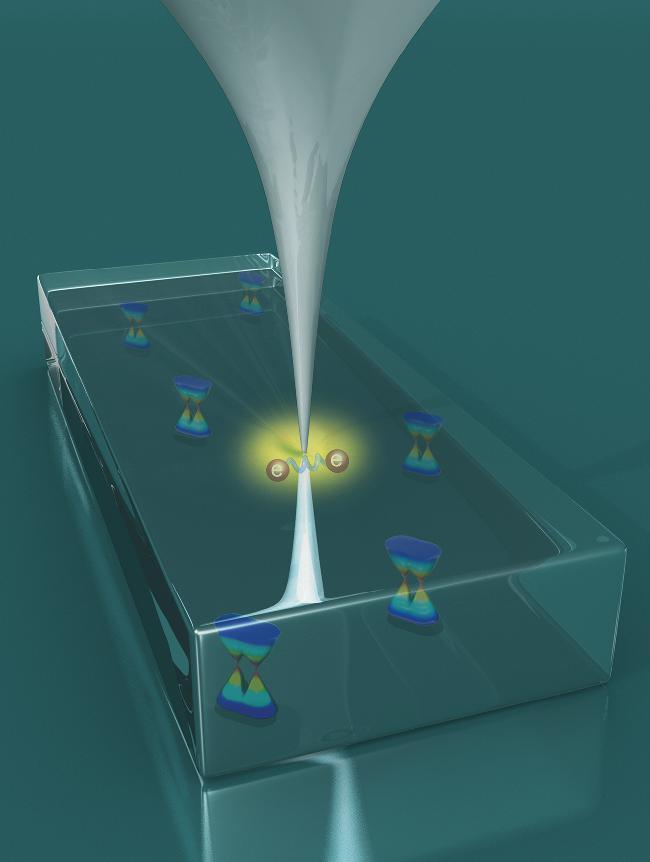
The schematic of tip-induced superconductivity in topological semimetals.

**Figure 2.**
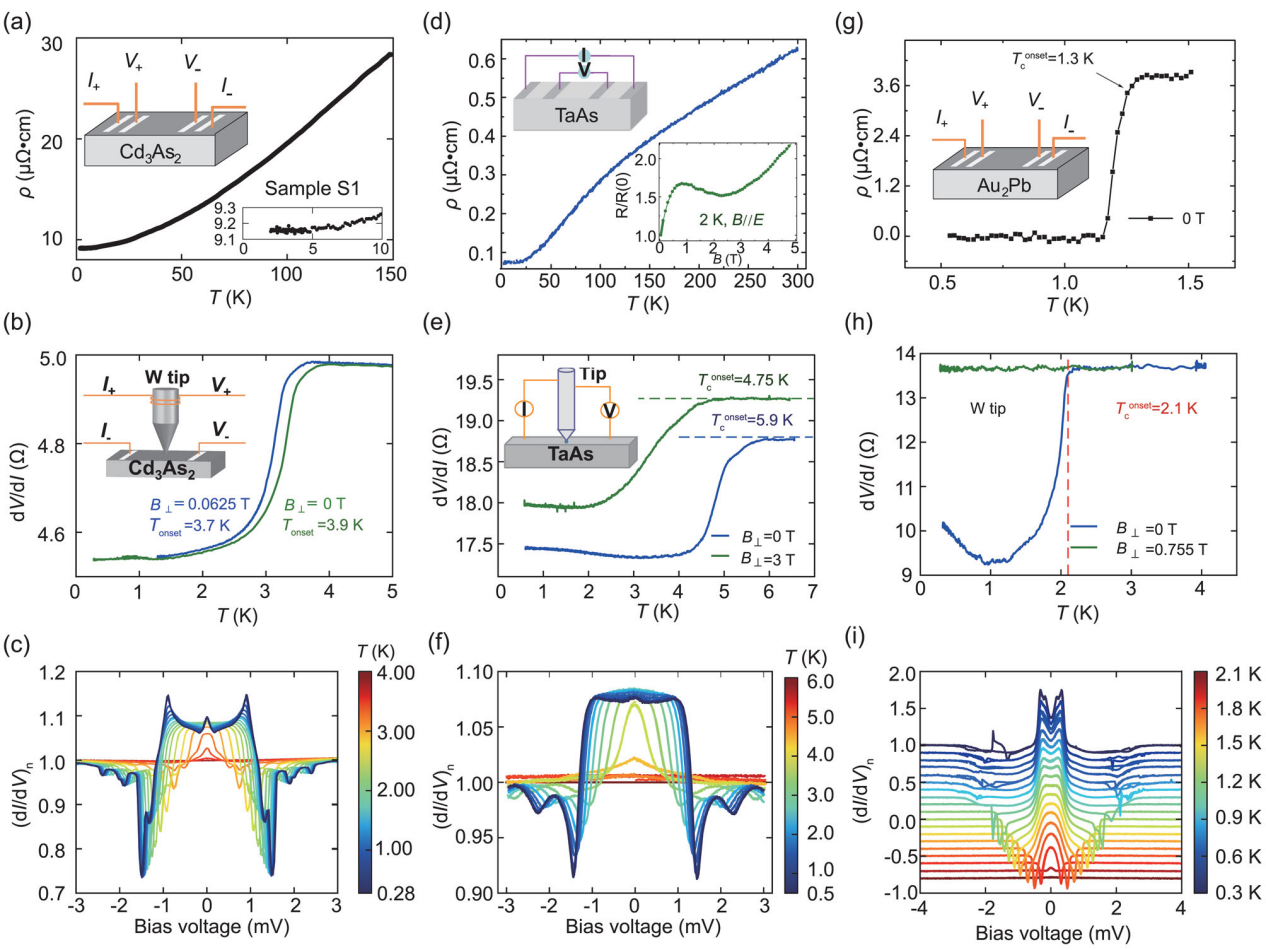
Tip-induced and enhanced superconductivity on topological materials. (a) Temperature dependence of four-probe bulk resistivity of Cd_3_As_2_ single crystal showing non-superconducting behavior. Upper inset: Schematics of the standard four-probe measurement configuration. Lower inset: Zoom-in of the resistance–temperature curve below 10 K. (b) Temperature dependence of the zero-bias resistance of the point contact (PC) measurement result on Cd_3_As_2_ single crystal with W tip showing superconductivity. Inset: Schematics of the PC measurement configuration. (c) Normalized dI/dV spectra of PC (PCS) on Cd_3_As_2_ at different temperatures without an external magnetic field. (d) Temperature dependence of four-probe bulk resistivity of TaAs showing non-superconducting property. Lower inset shows negative magnetoresistance as a signature of chiral anomaly when B//E. (e) and (f) Point contact measurements on TaAs with PtIr tip showing superconductivity. (g) Transport measurement of bulk Au_2_Pb single crystal showing T_c_∼1.3 K. (h) and (i) The zero-bias PC resistance as a function of temperature and the PCS at different temperatures for the point contact measurement of Au_2_Pb with W tip, showing an enhanced T_c_∼2.1 K. (a) to (c) Reprinted with permission from Wang *et al.* [16], Copyright 2016 NPG. (d) to (f) Reprinted with permission from Wang *et al.* [17]. (g) to (i) Reprinted with permission from Xing *et al.* [19], Copyright 2016 NPG.

Recent discovery of topological semimetals [9], such as topological 3D Dirac semimetals and Weyl semimetals, offers a new platform to look for and investigate topological superconductors if the materials can be superconducting. Differently from topological insulators [10,11], where only the surface state is topologically non-trivial and shows a linear energy-dispersion electronic structure, namely a Dirac cone structure [12], the bulk state of topological semimetals exhibits a topologically non-trivial Dirac or Weyl cone structure with relativistic quasiparticles [13,14]. In these topological materials, some intriguing quantum properties can be revealed, such as the discovery of log-periodic quantum oscillations [15]. Recently, by using non-superconducting metallic tips to carry out hard point contact measurements on non-superconducting Dirac semimetals Cd_3_As_2_ and Weyl semimetal TaAs crystals as shown in Fig. 1, it was found that the contact region becomes superconducting and point contact spectra (PCS) show ZBCP, symmetrical conductance peaks with dips combined in a structure, indicating the possibility of topological superconductivity [16,17] (Fig. 2a–f). Indeed, theoretically, there is a great chance that the induced superconductivity is topologically non-trivial. In previous studies, the hard point contact measurement was a common method to study superconductors. Now the technique has been demonstrated to be able to induce superconductivity in non-superconducting materials by using a non-superconducting tip as a modulation method like gating [18]. Actually, the tip for hard point contact measurement is also capable of enhancing the transition temperature (*T_c_*) for some superconductors, like Au_2_Pb [19] (Fig. 2g–i). Thus, the hard point contact measurement on topological semimetals, which also can be called the tip-induced superconductivity method here, offers a new way to trigger and detect topological superconductivity. Since neither the normal metal tip nor the sample is a superconducting material, tip-induced superconductivity on topological semimetals is reminiscent of the interface superconductivity in the LaAlO_3_/SrTiO_3_ (LAO/STO) system, which has not been seriously considered before. Therefore, further theoretical and experimental investigations are highly desired to understand what exactly happens at the interface and how to precisely analyse PCS. Besides, more evidence beyond the observed ZBCP and special PCS features in previous work is still necessary to completely demonstrate that the tip-induced superconductivity on topological semimetals is topologically non-trivial. Since topological superconductivity may show p-wave spin-triplet pairing symmetry, superconductivity is able to coexist with ferromagnetism, which can be experimentally tested.

Searching for intrinsic superconducting topological semimetals is another important direction in which to explore topological superconductivity. The type II Weyl semimetal MoTe_2_, where the Weyl cone is heavily tilted and Lorentz invariance is violated, has been reported as a superconductor at around 0.1 K [20]. The problem is that the *T_c_* is too low to carry out various experiments. By doping S into MoTe_2_, it has been found that the *T_c_* can be extremely enhanced and thus the superconductivity can be fully studied by transport, diamagnetic, heat capacity and scanning tunneling microscopy (STM) measurements [21]. Surprisingly, two superconducting gaps from the bulk state have been detected and the interband interaction is much stronger than the intraband interaction, which indicates s+– pairing. It is known that most Fe-based superconductors are s+– pairing. Nevertheless, differently from Fe-based superconductors, there are no magnetic orders in S-doped MoTe_2_. Thus, it would be a very interesting topic to figure out the mechanism of s+– pairing superconductivity in non-magnetic systems. Furthermore, it has been theoretically predicted that sign-changing superconductivity in the Weyl semimetal would form topological superconductivity [22]. On the surface of doped MoTe_2_, scanning tunneling spectroscopy (STS) studies reveal a much larger superconducting gap and the ratio of superconducting gap vs *T_c_* is much higher than that in weakly coupled BCS superconductors, which could result from parity mixing on the topological surface state. Another typical example of superconducting topological semimetal is TaIrTe_4_, where the surface superconductivity from Fermi arc states is detected while the bulk state is not superconducting [23]. It is noted that the ratio of superconducting gap vs *T_c_* of the TaIrTe_4_ surface is also much larger than the standard BCS ratio 3.53. Therefore, the extremely enlarged surface superconducting gap seems to be a universal property of topological superconductor candidates, which still needs further theoretical consideration and physical understanding.

Normally, the *T_c_* of discovered topological superconductor candidates is not high and most topological superconductor candidates are different from high-*T_c_* superconductors, where the strong correlation effect plays a great role. Low *T_c_* is a limitation for potential application in topological quantum computation. It is believed that the parent materials of many Fe-based superconductors are semimetal in type. Recent progress reveals that the surface states of some Fe-based superconductors can show the topological Dirac cone structure—a marriage between topology and high-*T_c_* superconductors [24]. Besides, in most studies on topological superconductors, a magnetic field has to be applied to detect Majorana-bound states, which is another practical limitation for application. However, very recently, without an applied magnetic field, Majorana-like ZBCP has also been detected on the top of Fe adatoms deposited by molecular beam epitaxy (MBE) on one-unit-cell-thick FeSe films on STO substrate, which are 2D high-*T_c_* superconductors with the *T_c_* higher than 50 K [25]. The quantum anomalous vortex nucleated at the magnetic ion in a strongly spin orbit coupled superconductor might induce the Majorana-bound state. The undoped one-unit-cell-thick FeSe film is considered a 2D Dirac semimetal. Therefore, it is highly desired to widely and deeply investigate the topological superconductivity and Majorana-bound states in high-*T_c_* Fe-based superconductors.

In summary, some topological semimetals show superconductivity at low temperatures and some are non-superconducting but can be modulated to be the superconductors, such as tip-induced superconductivity on topological semimetals. It is noted that the theoretical prediction points out hundreds of topological semimetals [26]. Moreover, the topologically non-trivial property has been predicted and observed in some Fe-based superconductors showing semimetal like electronic structure in undoped situations, which reveals a correlation between topological superconductors and high-*T_c_* superconductors and might pave the way to realizing feasible topological quantum computation in the future. Normally, high carrier density is required to achieve superconductivity, but the Fermi surface is small in semimetals when the Fermi energy is close to Weyl or Dirac points. Indeed, type II topological semimetals show higher carrier density and are easier to be superconducting. As for type I topological semimetals, superconductivity may originate from the topological surface state or doped bulk state. To fully demonstrate whether the superconductivity is topologically non-trivial, the Dirac physics contribution on the emerged superconductivity is necessary to be further clarified. Thus, the development of topological semimetals and other topological materials promises a great opportunity to detect and study topological superconductivity and will certainly stimulate the related investigations.
